# Efficacy and acceptability of S-adenosyl-L-methionine (SAMe) for depressed patients: a systematic review and meta-analysis of randomized controlled trials

**DOI:** 10.1192/j.eurpsy.2024.680

**Published:** 2024-08-27

**Authors:** N. Limveeraprajak, S. Nakhawatchana, A. Visukamol, C. Siripakkaphant, S. Suttajit, M. Srisurapanont

**Affiliations:** ^1^Faculty of Medicine Ramathibodi Hospital, Mahidol University, Bangkok; ^2^Faculty of Medicine; ^3^Department of Psychiatry, Chiang Mai University, Chiang Mai, Thailand

## Abstract

**Introduction:**

Current treatment options for depression remain unsatisfactory. SAMe, a naturally occurring body chemical available as a dietary supplement, was discovered in the 1950s. SAMe deficiency is associated with depression.

**Objectives:**

This systematic review and meta-analysis aimed to investigate the efficacy and acceptability of SAMe in treating patients with depression. The primary efficacy outcome was measured through the reduction in depression severity scores. All-cause dropout rates were assessed as indicators of treatment acceptability.

**Methods:**

To include the randomized trials comparing SAMe with other agents, we conducted a search on PubMed, Embase, and the Cochrane Library from their inceptions until April 27, 2023. The quality of trials was assessed using version 2 of the Cochrane risk-of-bias tool for randomized trials (RoB 2). Depression severity and overall dropout rates were synthesized using a random-effect model for frequentist pairwise meta-analysis.

**Results:**

We categorized 23 trials (N = 2,234) into 11 trials comparing SAMe vs. placebo, 5 trials comparing SAMe + antidepressant vs. placebo + antidepressants, and 7 trials comparing SAMe vs. antidepressants. SAMe demonstrated a significantly greater reduction in depressive symptoms compared to placebo (SMD = -0.58, 95%CI [-0.93; -0.23], I2 = 68%), as can be seen in Figure 1. A trend was observed wherein SAMe showed a lesser reduction in depressive symptoms compared to antidepressants (SMD = 0.06, 95%CI [-0.06; 0.18], I2 = 49%). When administered alongside ongoing antidepressant treatment, SAMe did not significantly differ from placebo in reducing depressive symptoms (SMD = -0.16, 95%CI [-0.44; 0.13], I2 = 57%). In the subgroup analysis of 11 trials comparing SAMe and placebo, it was found that while the intramuscular (SMD = -0.92, 95%CI [-1.39; -0.44]) and oral routes (SMD = -0.66, 95%CI [-1.24; -0.08]) revealed the efficacy of SAMe, the intravenous route did not exhibit the same efficacy (SMD = -0.16, 95%CI [-0.47; 0.14]). The efficacy of SAMe was not influenced by factors such as physical illness, history of antidepressant nonresponse, proportion of females, age, duration and dosage of SAMe supplementation, publication year, and baseline depression severity. There was no significant difference in dropout rates between SAMe and controls.

**Image:**

**Figure:**
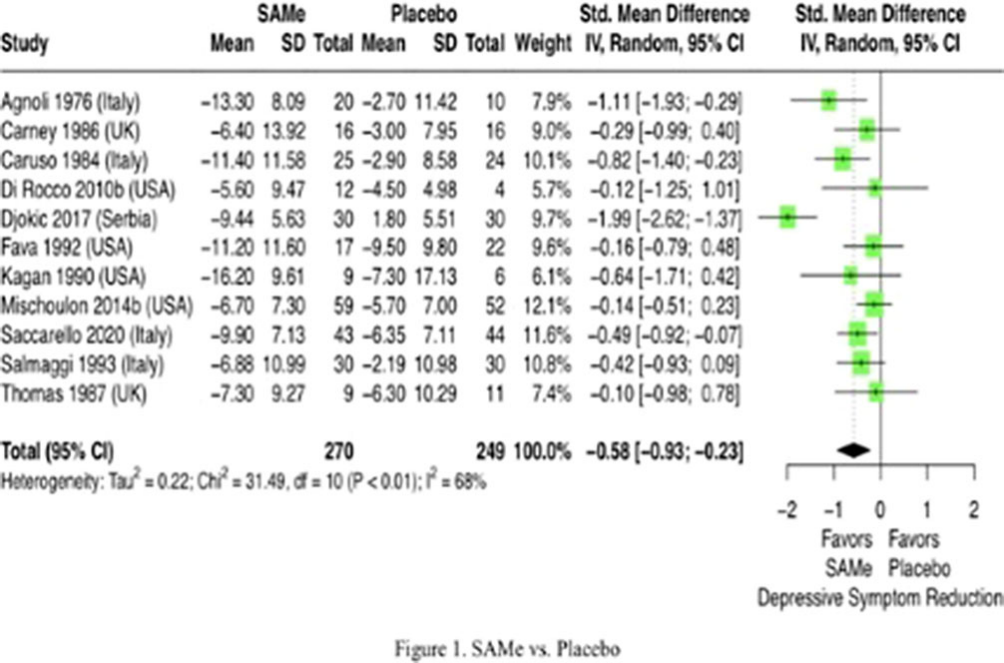

**Conclusions:**

Limited evidence suggests that SAMe is well accepted and effective in reducing depressive symptoms. However, its antidepressant effect may not be as strong as that of traditional antidepressants. Randomized-controlled trials comparing SAMe to antidepressants in depressed patients, both with and without ongoing antidepressant use, are still necessary.

**Disclosure of Interest:**

None Declared

